# Bladder outlet obstruction and non-invasive urodynamics - The future?

**DOI:** 10.4103/0970-1591.45529

**Published:** 2009

**Authors:** Nitin S. Kekre

**Affiliations:** Department of Urology, Christian Medical College, Vellore - 632 004, Tamilnadu, India E-mail: editor@indianjurol.com

Let me first wish you all a very happy, prosperous, and peaceful 2009. The USICON 2009 at Indore was good a meeting, especially the scientific content and ofcourse the wonderful Indore food and hospitality.

This issue has a symposium on a subject that is very close to my heart. All of us desire of noninvasive diagnostic tests and minimally invasive therapies. Conventionally BOO is diagnosed by pressure-flow (PF) studies. PF studies are gold standards but are not patient friendly. They are invasive, require catheterization and are performed under unphysiological conditions. Would it be feasible to make the diagnosis of BOO without invasive PF studies? Free flow rate, bladder wall thickness, symptoms score etc have been used alone or in combination to diagnose BOO. But none of them can replace PF studies. The non-invasive means of measuring bladder pressure and to correlate with flow would be the obvious way forward. Mr. Rob Pickard, Senior Lecturer at Freeman Hospital, Newcastle, Upontyne, has guest edited this symposium on *Role of Noninvasive Urodynamics* in BOO. The Department of Urology and Medical Physics at Freeman Hospital has made significant contribution in the field of urodynamics, and their work on ambulatory urodynamics and noninvasive urodynamics is well known. I am indebted to Rob for agreeing to do this symposium for IJU. He and his colleagues have done a wonderful job and I am sure all of you who have interest in the field of urodynamics would enjoy reading this symposium.

This issue comes with some very important review articles. Venous thromboembolism is no longer a Western problem; it is not uncommon in Indian population and we encounter them regularly in our practice. Dr. Sunil Agarwal *et al*, the consultant vascular surgeon from Christian Medical College, Vellore, have provided indepth review and detailed current recommendations. Vincentini *et al* and Symons *et al* have elaborately reviewed the current status of endoscopic surgery in renal stone disease and PUJ obstruction. Professor G. Läckgren provides insight into the current endoscopic treatment of VUR.

The IJU symposium on medical writing during Indore meeting was well received and it was heartening to note that many of you have shown interest in medical writing. To improve the standards of medical writing, the editorial team of IJU is planning to conduct workshops on medical writing and peer review. I am happy to inform that the council of USI has approved this program. Dr. Anil Mandhani will be hosting first workshop on medical writing at SGPGI, Lucknow. The dates for this workshop will be advertised in Urology News Letter and IJU. Those interested in attending this workshop can contact Dr. Anil Mandhani directly at SGPGI as the number of registration will be limited to 25 only. I am happy to inform that all Postgraduates who are registered as Associate Members with USI would start getting the hard copy of the journal.

To improve and encourage quality submission to IJU, it has been decided to introduce 2 best paper awards. These awards would be announced before the annual meeting and the prize will be given at a special function, during the USI annual meeting. I would encourage you all to submit quality papers to IJU and win the IJU gold medal.

Once again my sincere request to send constructive criticism and I shall try my best to incorporate your suggestions

With best wishes.

